# ZnO Electrodeposition Model for Morphology Control

**DOI:** 10.3390/nano12040720

**Published:** 2022-02-21

**Authors:** Javier Orozco-Messana, Rubens Camaratta

**Affiliations:** 1 Institute for Materials Technology, Universitat Politecnica de Valencia, 46022 Valencia, Spain; 2 Center for Technological Development (CDTec), Universidade Federal de Pelotas, Pelotas 96010-610, RS, Brazil; rcamaratta@ufpel.edu.br

**Keywords:** ZnO, nanorods, electrochemical modelling, morphology control

## Abstract

In this research, a model for electrodeposition of zinc oxide (ZnO) nanostructures over indium-doped tin-oxide (ITO) glass using pulsed current and zinc chloride as source of zinc was proposed. For the model, reactions kinetics rate constants were evaluated by obtaining the reaction product solid mass of the various species through time using an electrochemical quartz crystal microbalance (EQCM). To obtain a mathematical model of the electrodeposition using Ansys CFX 2D simulation software, the reaction kinetics rates were used to calculate mass transfer in the volume closest to the surface. The model was applied to the experimental electrodeposition conditions to validate its accuracy. Dense wurtzite nanostructures with controlled morphology were obtained on a indium-doped tin-oxide (ITO) glass. Sample characterization was performed using high-resolution field emission scanning electron microscopy (FESEM) and transmission electron microscopy (TEM) on focused ion beam milled (FIBed) sheets from wurtzite mono-crystals. Average crystallite size was evaluated by X-ray diffraction (XRD) using the Scherrer equation, and superficial areas were evaluated by Brunauer, Emmett, and Teller (BET) method. Through the experimental results, a chemical model was developed for the competing reactions based on the speciation of zinc considering pH evolution, and kinetic constants, on the oxygen rich aqueous environment. Owing to the model, an accurate prediction of thickness and type of electrodeposited layers, under given conditions, is achieved. This allows an excellent control of the optical properties of Wurtzite as a photon absorber, for an efficient separation of the electron-hole pair for conduction of the electric charges formed. The large surface area, and small wurtzite crystallites evenly distributed on the thin film electrodeposited over the ITO conductive layer are promising features for later dye-sensitized photovoltaic cell production.

## 1. Introduction

Zinc oxide, when in wurtzite crystalline form, presents very relevant optical properties in the visible spectrum. When considering photovoltaic applications, its semiconductor bandgap (3.33 eV), together with a high binding energy of (~60 meV) at 20 °C allows very relevant photon absorption [1]. It exhibits outstanding chemical stability, favoring photo-corrosion resistance, at a very low cost. These properties make ZnO a perfect candidate for its industrial use in photocatalysts [2], ultraviolet (UV) detectors [3], light-emitting diodes [4], and dye-sensitized solar cells (DSSCs) [5]. Careful control of wurtzite surface area, distribution, and orientation, allows conduction control for electron-hole pairs, and excellent transmittance in the visible spectral region. ZnO samples were produced by electro-deposition using alternate current [6] for a better understanding and control of the speciation reactions taking place depending on pH and ion concentrations [7]. Homogeneous thin films can be obtained perfectly tuned to the application desired. These nanostructures can be further tuned to the desired application by doping and defects control [8]. Recent introduction of solid-state molecules [9] forecast an excellent future for these ZnO nanostructures.

ITO back contacts are the traditional option for (semi)transparent photovoltaic devices and have relevant advantages for the new semiconductors based on ZnO and solid-state polymers [10]. However, market drive toward low-cost photovoltaics is reducing the use of ITO as a transparent electrode, first due to the expensive vacuum sputtering process required for preparing these electrodes [11], together with the strategic availability of indium (only 16,000 tons in earth [12]). Therefore, new generation of commercial devices have reduced dramatically the use of ITO for cost effective polymer solar cells. New (semi)transparent back-contacts are introducing ZnO as an option [13] but losing some efficiency on charge separation. However, an extremely thin ITO layer (also recovered from other solar cells) can be upgraded by controlled electrodeposition of ZnO in practical applications [11,14]. ZnO serves as polymer support, also enhancing the mobility of photoexcited electrons [14].

Wurtzite structural defects on ZnO can control electron mobility and light absorption in photovoltaic devices. Bulk defects and microstructural grain orientations, together with boundary surface states (especially those below the conduction energy band) inhibit electron mobility due to competing recombination [15]. Therefore, a well-controlled nucleation/growth evolution leads to excellent performance of these semiconductor junctions [15].

Particle’s morphology can enhance effective semiconductor surface producing a very large active surface for charge separation, improving photovoltaic efficiency when combined with the previously mentioned electron transport behavior. Understanding the variables controlling the morphology of nanostructure ZnO will allow a relevant increase in photovoltaic efficiency [16].

## 2. Materials and Methods

### 2.1. ZnO Electrodeposition Model Development

For studying the controlling elements for nucleation and growth, the model considers the speciation reactions responsible for both processes. There are four possible reactions on the relevant speciation route, and according to [7] we can consider that all happen at the same time within oxygen-saturated aqueous solution, for low concentrations, and slow deposition rates (all verified in our system):

$${\mathrm{Zn}}^{2+}+{\mathrm{OH}}^{-}\overset{K_{\mathrm{OH},1}}{\to }{\mathrm{ZnOH}}^{+}+{\mathrm{OH}}^{-}\overset{K_{\mathrm{OH},2}}{\to }\mathrm{Zn}{\left(\mathrm{OH}\right)}_{2}+{\mathrm{OH}}^{-}\overset{K_{\mathrm{OH},3}}{\to }\mathrm{Zn}{\left(\mathrm{OH}\right)}_{3}^{-}+{\mathrm{OH}}^{-}\overset{K_{\mathrm{OH},4}}{\to }\mathrm{Zn}{\left(\mathrm{OH}\right)}_{4}^{2-}$$

All reactions are happening at the same time with different reaction rates (K_i_) which have been studied experimentally in [17]. Analyzing only the hydroxides speciation the pH is the controlling variable regarding zinc availability for electrodeposition, or alternatively, producing relevant speciated hydroxides.

Reaction kinetics must be considered now to evaluate the production of the various species through time. According to the differential rate law a given reaction “x” yields a given rate following:

$$r_{x}=k_{x}\underset{i}{\prod }c_{i}^{n_{xi}}$$

where *k_x_* is the rate constant for reaction x, *c_i_* is the concentration of reactant *i*, *n_xi_* is reaction order in reaction x for reactant *i*, and *r_x_* is the reaction rate (mol/s) for a given (stable) set of reaction conditions (in our case constant temperature *T*, and oxygen saturation). Under these conditions *k_x_* is stable and can be evaluated experimentally. Reactants’ reaction order has already been evaluated in [17] for the Zn^++^ system.

The model proposed for the research uses the set of equations presented in Table 1. Model parameters are evaluated on the following points, after an analysis of their relevance, and allowing the calculation of the optimal conditions for nucleation and growth according to the desired morphology.

### 2.2. Experimental Determination of ZnO Electrodeposition and Simulation Model

**Figure 1:**
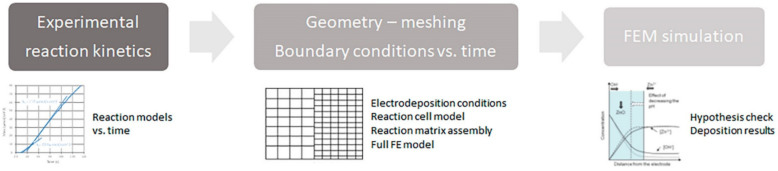
Proposed simulation process.

For quantifying the proposed chemical model (first stage in Figure 1), the reaction rate constants must be determined experimentally according to [18]. The procedure includes obtaining the actual reaction product solid mass obtained for each reaction. This case was evaluated using an Infinicon IPN603800 Research Quartz Crystal microbalance (East Syracuse, NY, USA).

The experimental setup included a calibrated quartz crystal prepared with a conducting layer of sputtered ITO thin film (7 Ω/sq) and the optimal conditions for the electrodepositions (taken from [6]). A solution with pH at 6, and with a temperature kept thermostatically constant at 60 °C, containing the following electrolytes was used: 0.1 M KCl (Rectapur©, Leuven, Belgium, purity >99%), ZnCl_2_ 5·10^−3^ M (Panreac, Barcelona, Spain, purity >98%), and continuous oxygenation by a bubbling flow of 0.1 l/min of commercially pure oxygen. The electrochemical experiments were performed potentiostatically in a 3-electrodes electrochemical cell with the substrate as cathode, a Pt sheet as counter electrode, and an Ag/AgCl electrode (SE) as the reference electrode (VSE = 0.2 v).

For obtaining the reaction rates, a current step (−4 mA) was applied and the mass deposited is recorded with time.

With this information, the simulation model proposed in Figure 1 can be completed following all the items in the second step to calculate mass transfer in the volume closest to the surface (step 3 in Figure 1). Calculations were done using a simple finite element model using Ansys CFX 2D v12.0 [19]. The geometry considered includes only the rectangle from the electrodeposited surface to the platinum counter electrode. The mesh has a high resolution near the electrodeposition plane to resolve boundary layer (mass transfer of Zn^++^ and other species in solution), the turbulence shear stress transport (SST) model was selected for fluid movement. The fluid is single phase water, with all speciated ion concentrations as variables except those which, due to their high concentration, can be considered constant (OH^−^, Zn^++^). Volumetric expansivity coefficients for solid species, as well as viscosity for water at 60 °C, are considered constant. Electrical boundary conditions (constant nominal current) are applied through time on active surfaces to determine deposition rates on the sample surface.

This mathematical model will be applied to the experimental electrodeposition conditions to validate its accuracy.

### 2.3. ZnO Electrodeposition

Electrodeposition-pulsed current was programmed using an Autolab potentiostat (PGSTAT 302N, Utrecht, the Netherlands) using the NOVA v2.0 software (Autolab, Utrecht, the Netherlands). A conventional three-electrode glass cell using a working electrode (ITO sputtered sample) with an open 1 cm^2^ circular surface, as well as a counter-electrode (Pt foil), and a reference electrode (Ag/AgCl in saturated KCl) was used.

Five ceramic substrates per set of working variables covered with an ITO-sputtered layer (resistivity at 25 °C 10 Ω/cm^2^) were used. Later, samples were ultrasonically cleaned in a mixture of distilled water with liquid neutral soap for 10 min. All samples were rinsed for 10 min in distilled water. The last step included immersion in isopropanol for 10 min for later drying with a nitrogen current.

From the application of the computer model, each electrodeposition cycle included two periods:

- A period of constant current (−4 mA).
- A period for concentration homogenization (no current applied).
- Then, the electrodepositions were carried out in two phases.
- The first phase of 70 s with cycles of 1.5 s of constant current and 0.5 s without current.
- The second phase of 730 s with cycles of 1 s of constant current and 1 s without current.

Samples were produced with times ±5, 10, and 15% starting from the optimal prediction from the model. All samples were characterized later. 

### 2.4. ZnO Characterization

High-resolution field emission scanning electron microscopy (HRFESEM) performed with a Zeiss Gemini SEM500 (Oberkochen, Germany) was used for morphological analysis. No preparation was required for the samples except a colloidal silver compound Electrodag 1415M from SMS which creates a consistent rigid layer protecting the sample for fibbing. 

**Figure 2:**
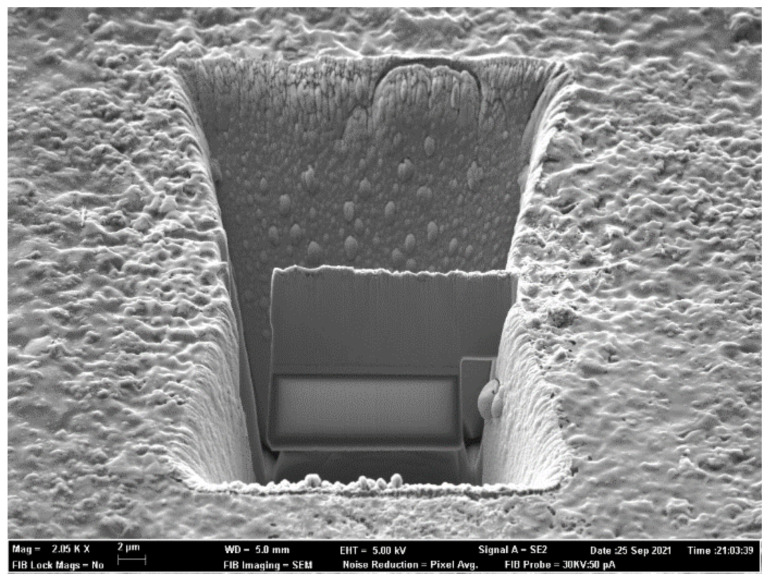
Lamella fibbed from sample.

The selected sample (one per set of working conditions) was prepared by fibbing (with field emission scanning electron microscope, FESEM, Zeiss Auriga Compact, Oberkochen, Germany) for obtaining a very thin lamella (Figure 2) later thinned by ion milling with a Fischione Instruments 1010 (Pittsburgh, PA, USA) for transmission electron microscopical (TEM) observation using a 200 kV Jeol JEM-2100F (Akishima, Japan). Point composition was determined with a Digistar AT3D X-ray diffraction equipment from Oxford (Abingdon, UK) for obtaining spot diffraction patterns.

In order to measure surface area, and crystallite sizes, a Brunauer, Emmett, and Teller (BET) surface area analysis was performed with a gas adsorption instrument Autosorb Quanta chrome model NOVA 1000 (Boynton Beach, FL, USA).

## 3. Results and Discussion

### 3.1. ZnO Electrodeposition Model 

Existing electrochemical models typically consider only dissolved precursors (mostly in ionic form) [20] and controlled by redox potentials. Later, mechanistic modelling [21] has demonstrated the role of all zinc speciation alternatives, and their corresponding reaction kinetics, controlled by pH and temperature.

Our theoretical approach is based on an aqueous system, with a starting pH of 6, completely saturated with oxygen during the whole reaction (oxygen bubbling continuously). The elemental stages for zinc oxide electrodeposition follow two competing reactions, first reduction to produce hydroxide, followed by zinc ions combining with the hydroxide for ZnO electrodeposition:

$$O_{2}+2H_{2}O+4e^{-}\to 4{\mathrm{OH}}^{-}$$

$${\mathrm{Zn}}^{2+}+2{\mathrm{OH}}^{-}\to \mathrm{ZnO}+H_{2}O$$

The most thermodynamically stable form of solid ZnO is the wurtzite (HC) crystal. Depending on preferred directional growth, its shape can vary from a thin plate to slender columns. Different morphologies can be obtained combining nucleation and directional growth. Other complex shapes (flower-like) are also possible when wurtzite cannot be formed due to a lack of thermodynamic equilibrium conditions.

Density of crystals depends on nucleation rate, and their length is controlled through equilibrium growth. The desired morphology for photovoltaic applications depends on well-aligned monocrystalline wurtzite thin columns (rods).

As identified in [6], initial conditions produce an amorphous Zn(OH)_2_ followed by an instantaneous nucleation of ZnO once the electrodeposition voltage is reached (due to oxygen saturation). Electrodeposition occurs only from Zn(OH)_2_ according to [6]:

$$\mathrm{Zn}{\left(\mathrm{OH}\right)}_{2,\left(aq\right)}\to \mathrm{ZnO}+H_{2}O$$

Zn(OH)_2_ appears after immersion of the clean conductive surface producing a compact amorphous layer on the electrodeposition substrate [6]. Once electrodeposition starts, (current or voltage controlled) ZnO is nucleated (and grows) over this amorphous layer depending on the relative concentration of Zn(OH)_2_. 

This completes the basic rate for the precipitation reactions which are competing among themselves.

Electrodeposition is an electrically driven process happening mainly at the conductive surface and with no relevant impact on ion concentrations on solution for thin films deposition. In the following paragraphs, we will evaluate the relevance of each reaction described in Table 1.

Reaction 1 is independent from electrodeposition and will be considered in equilibrium all through the process since it does not affect surface reactions. It provides a stable average concentration of reaction species and does not influence other reactions progress due to the relatively stable concentration in the driving components.

When considering the physical electrodeposition system and the experimental evidence detected in [6], Zn(OH)_2_ appears only on conductive surfaces during electrodeposition, therefore reaction 4 is the only one active until the sample surface is completely saturated with Zn(OH)_2_. Its constant (*k*_4_) can be calculated by the initial mass vs time deposition slope and nuclei occupy less than a hundredth of the Zn(OH)_2_ surface [6].

On the other hand, ZnO crystals nucleate over this hydroxide (reaction 5) making all other reactions (2, 3, and 4) irrelevant during the initial nucleation process. Reaction 3 controls hydroxide formation.

Growth depends on reaction 2. Its progress requires ZnOH^+^ in the solution on nucleated spots since its concentration controls ZnO growth. The rate constant for this reaction (*k*_2_) can be evaluated from the slope on the stable part of the deposition curve (deposited mass vs. time).

**Figure 3:**
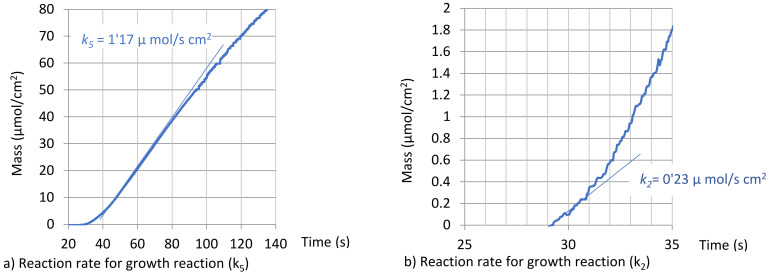
Experimental determination of reaction rates for nucleation (slope of the initial part of the curve, *k*_2_) and growth (stable slope during growth, *k*_5_) since all other reactions are negligible (*k*_1_ = *k*_3_ = *k_4_* = 0).

The curves obtained are presented in Figure 3.

The model is complete and allows a practical procedure for controlling both nucleation and growth through a pulsed current approach that should avoid depletion of the active species (ZnOH^+^) over the sample surface during both electrodeposition phases having in mind that roughly nucleation is nearly one order of magnitude bigger than growth but requires less than a hundredth of the nucleation mass per unit surface.

Electrodepositing at a constant voltage level does not allow good control of surface speciation and its concentration for thin films. Constant current electrodeposition can provide an even and homogeneous layer but not completely dense. As explained before, morphology control depends on ion availability at the right place and moment.

Initial experimental studies developed by Reyes-Tolosa et al. [6] show experimental evidence of good morphology results (nanocolumns) for ZnO in wurtzite form being deposited by a galvanostatic pulsed current of −4 mA maximum value and 0.5 cycles per second. Each cycle included a pulse of continuous current for 1 s followed by another 1 s part with no current injection. This demonstrated experimentally a morphology with excellent optical properties.

From the application of the computer model described in 2.2, the energy required to deposit a 20-nm layer of Zn(OH)_2_ is applied on 1.5 s (at −4 mA constant current). During that time the concentration of driving ions (ZnOH^+^) is depleted to around 60% in the limit layer over the deposition surface making other competing reactions dominant. The active part of the cycle must then be stopped until the concentration becomes homogeneous again (0.5 s according to the model). A new cycle can then begin.

After 70 s (or 35 cycles) the Zn(OH)_2_ layer reaches theoretically 700 nm; the dominant reaction is ZnO nucleation. The cycle optimal conditions from the model are now (with the same −4 mA current) 1 s active period followed by 1 s stop period for nucleation. This develops a very dense homogeneous nucleation before the unavailability of Zn(OH)_2_ makes growth dominant. This is achieved after 50 s (or 25 cycles) and then growth begins in all nucleated sites at a rate of 1.4 nm (cumulative nuclei height) per cycle (same cycle conditions). Key results obtained from the simulation model are shown in Table 2.

Zn(OH)^+^ concentration controls the first deposition phase producing a very compact and homogeneous Zn(OH)_2_ layer acting as perfect seed surface for wurtzite ZnO nucleation. Reducing the concentration of the controlling species changes on the surface resistance (due to the hydroxide layer) launches the second phase of the electrodeposition which develops preferentially a dense and uniform layer of ZnO seeds which begin to grow once the hydroxide layer is not accessible by the controlling species. Now growth is maintained and should be stopped when the adequate height of the wurtzite columns reaches its optimal value.

### 3.2. ZnO Characterization

**Figure 4:**
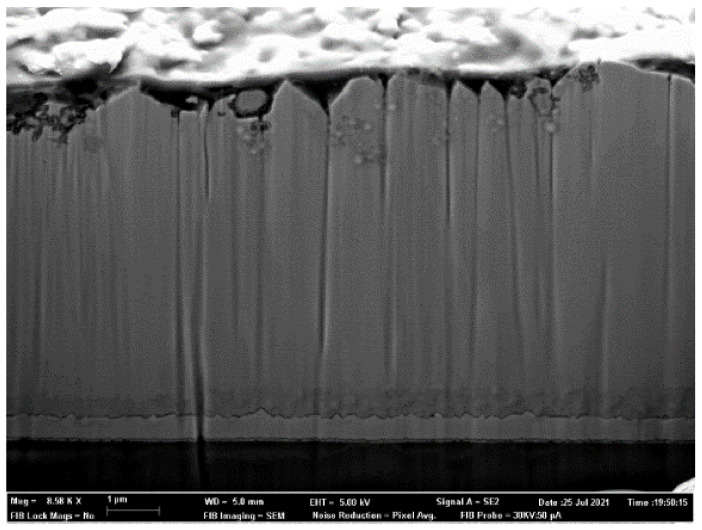
Optimal ZnO electrodeposited morphology.

Morphological variations followed the expected pattern validating the computer model predictions. In Figure 4, the morphology can be observed for the sample prepared exactly with the pulsed current optimized with the model.

Three samples from the optimal morphology set were studied rendering equivalent results. As previously stated, they were ion milled and their study was carried out on TEM for identifying the phases and their crystallinity through spot diffraction patterns.

**Figure 5:**
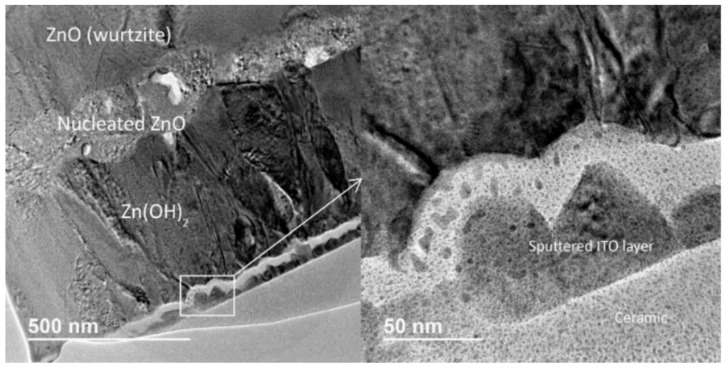
Transition sequence for electrodeposited ZnO wurtzite with magnified detail.

In Figure 5, a TEM micrograph of the transition area from the amorphous Zn(OH)_2_ layer to the wurtzite ZnO, through the ZnO nucleation layer is presented. In the lower magnification image (left) the sputtered ITO layer can be identified (with a clear detail on the higher magnification detail shown). Directly on top of the ITO layer the amorphous Zn(OH)_2_ layer acts as a precursor to the ZnO nucleation later growing into wurtzite columns. 

**Figure 6:**
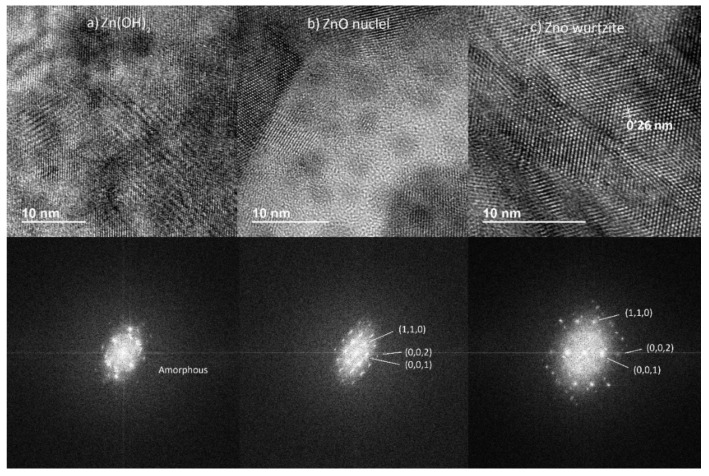
Electrodeposited phases and corresponding X-ray (XR) diffraction patterns for: (**a**) Zn(OH)_2_; (**b**) ZnO nuclei; (**c**) ZnO in wurtzite form.

In Figure 6, HRTEM images and corresponding ring diffraction patterns are presented. Selected spots from the previous sample allow the identification of the main microstructural elements on the ZnO layer presented before in Figure 5:

(a) Random microstructure for amorphous Zn(OH)_2_ with corresponding irregular diffraction pattern below.
(b) Small ZnO wurtzite monocrystalline nuclei with high quality XR diffraction pattern below showing main crystal directions (1,1,0), (0,0,1), (0,0,2).
(c) Average quality crystalline microstructure for a ZnO wurtzite column showing the lattice spacing (0.26 nm). Below the expected average quality diffraction pattern (with main crystal directions (1,1,0), (0,0,1), (0,0,2)).

After validating the expected morphology from our computer model, and the associated crystalline microstructure on the thin film, an evaluation of the semiconducting properties of the sample is required to check its performance.

**Figure 7:**
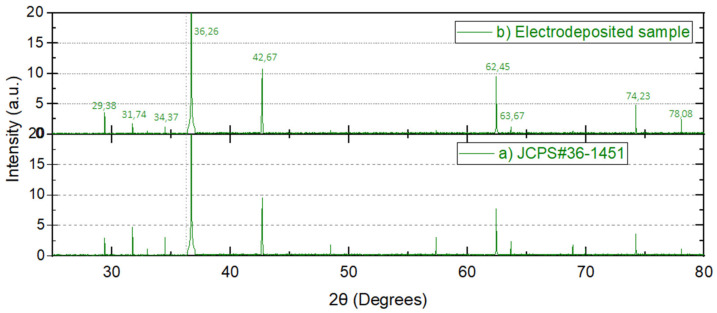
X-ray diffraction spectra for: (**a**) pure ZnO [23]; (**b**) electrodeposited ZnO powder.

**Figure 8:**
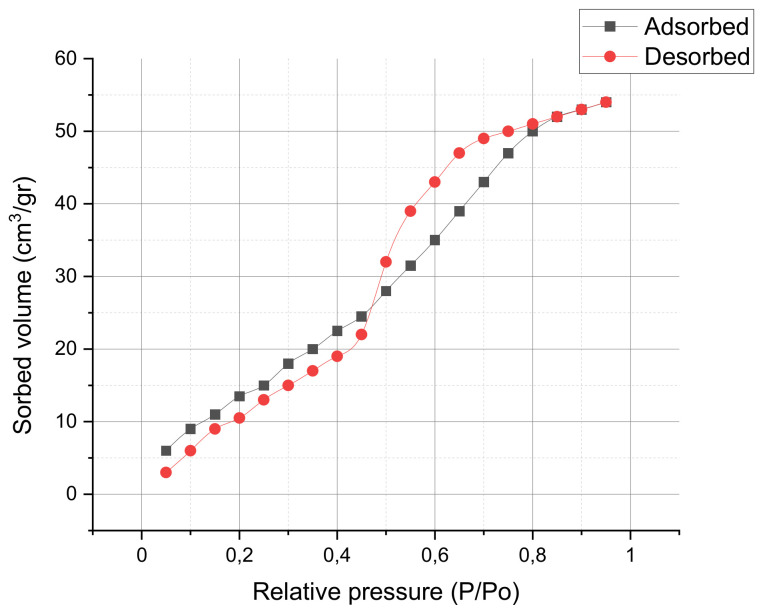
BET isotherms of electrodeposited sample after extraction.

According to Sedlak et al. [22] for ZnO nanoparticles, as the high electron diffusion coefficient (D) increases, the electron recombination lifetime (τ) decreases with increasing crystallite size up to 32 nm. Therefore, the crystallite size should be well below 32 nm with a surface area above 15 m^2^/gr for efficient thin-film photovoltaic applications. In Table 3, the experimental results obtained on shaved powder obtained from the optimal ZnO electrodeposited layer (using diffractogram in Figure 7 and the Brunauer–Emmett–Teller (BET) curve in Figure 8) are presented. Since the actual surface area (20.6 m^2^/gr) exceeds significantly the 15 m^2^/gr threshold with a crystallite size of 10.2 nm (below 32 nm), the electron recombination lifetime is maintained at reasonable levels. The ZnO electrodeposited thin-films obtained are adequate for efficient photovoltaic applications and the proposed model renders good results for electrodeposited thin-film layers.

In Figure 7 the X-ray diffraction pattern (XRD) of electrodeposited powder presents the characteristic wurtzite structure peaks (as can be checked from the overlaps on spectra “a” and “b”). When comparing card JCPDS#36-1451 [23] with the electrodeposited sample, the strongest peak observed at 36.26°, corresponding to plane (101), ensures a strong crystallinity due to its tiny full width half maximum.

The analysis on the BET 600 °C isotherm curves in Figure 8 offers a porosity of 64.8 m^2^/gr with an average pore size of 26.31 Å. The shape presented by the isotherm curves in Figure 8 when compared with the classical 5 isotherm types observed by Brunauer, Deming and Teller, correspond in both cases to type V. Type V curves present small adsorbate–adsorbent interaction potentials presenting pores in the 1.5–100 nm range.

**Figure 9:**
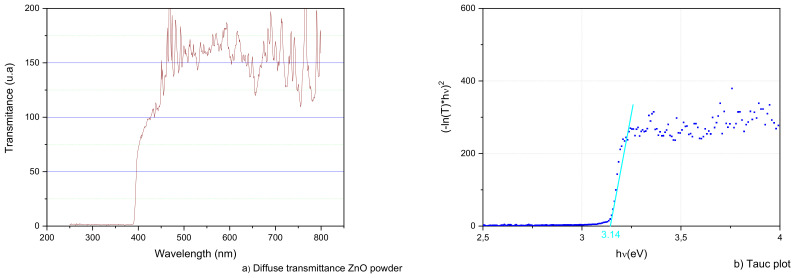
(**a**) Diffuse transmittance spectra of electrodeposited ZnO powder, and (**b**) Tauc plot for bandgap calculation.

As a final check on the optical properties of the electrodeposited ZnO powder the diffuse transmittance spectra plot for the optimal ZnO sample is shown in Figure 9. The transmittance analysis shows the band gap onset in the 450–500 nm region.

From the diffuse transmittance spectrum in Figure 9a, the Kubelka–Munk remission function is used to calculate the sample bandgap obtaining 3.14 eV (graphical extrapolation can be observed in Figure 9b) [24]. Therefore, the electrodeposited ZnO can absorb a broad range of wavelengths of visible light as can be checked in [25].

After the validation evidence from all the characterization checks, the simulation model allowed the designing of a high-quality electrodeposited thin film presenting a dense columnar array of ZnO crystals in wurtzite form aligned on the optimal (0,0,2) direction. The quality of the thin-film relates to excellent crystallinity, without excessive defects, and morphology parameters (surface area, crystallite size) matching the required recombination values and electron mobility for an optimal photovoltaic application.

## 4. Conclusions

As analyzed in this paper, when electrodepositing ZnO, from ZnCl_2_ and KCl, final surface morphology can be predicted through the proposed speciation computer model. This model is based on the kinetical analysis of all chemical speciation routes possible for ensuring a direct control on nucleation and growth of nanocrystals. ZnO nano-crystal evolution was designed through a pulsed current control cycle tuned for ensuring dense and homogeneous nucleation followed by homogeneous growth of the wurtzite nano-columns.

The kinetical determination of competing speciation reactions has been validated through experimental evidence explaining nano-crystal evolution during the electrodeposition process.

After running the computer model for ZnO electrodeposition, experimental evidence validates the 2-phase electrodeposition for the chemical speciation prediction.

It was demonstrated that nucleation starts over a fast-growing Zn(OH)_2_ amorphous layer where ZnO particles nucleate faster when ZnOH^+^ ion concentration in solution is not depleted (as the evolution on the simulation model suggests). Growth takes over once no further nucleation sites are available ensuring dense high-quality nanocrystals tailored to photovoltaic applications.

Additionally, experimental characterization from the ZnO microstructure provides a dense and homogeneous surface with wurtzite crystals uniformly distributed with small average crystallite sizes and high surface area. The pulsed controlled electrodeposition improves the quality of the surface morphology of the film.

The high-quality thin films produced, after an optimization process which should include doping for fine tuning the desired bandgap according to the final semiconductor desired, will allow green routes for producing building integrated solar cells using advanced techniques such as inkjet printing on multiple surfaces for enhanced power conversion efficiency on inkjet-printed cells with more advanced designs [26,27].

The development of these low-cost thin films introduces a simple, cheap, and green route for new generations of durable photovoltaic materials which can add energy generation as a new functionality to many industrial materials. 

As a final summary, the design of pulsed current electrodeposition with the proposed computer model based on speciation chemical information allows an excellent procedure for producing high quality oxide thin films to be used in semiconducting applications.

## Figures and Tables

**Table 1:** Speciation differential rate equations.

x	Reaction	*k_x_*	*n_xi_* (*i* = 1.2)		Deposition Reaction Rate
1	${\mathrm{Zn}}^{++}+{\mathrm{OH}}^{-}\to {\mathrm{ZnOH}}^{+}$	$k_{1}$	*1.1*	*0.4*	$k_{1}C_{{\mathrm{Zn}}^{++}}^{n11}C_{{\mathrm{OH}}^{-}}^{n12}$
2	${\mathrm{ZnOH}}^{+}+{\mathrm{OH}}^{-}\to \mathrm{ZnO}+H_{2}O$	$k_{2}$	0.6	*0.5*	$k_{2}C_{{\mathrm{ZnOH}}^{+}}^{n21}C_{{\mathrm{OH}}^{-}}^{n22}$
3	${\mathrm{Zn}}^{++}+2{\mathrm{OH}}^{-}\to \mathrm{Zn}{\left(\mathrm{OH}\right)}_{2}$	$k_{3}$	0.9	*0.8*	$k_{3}C_{{\mathrm{Zn}}^{++}}^{n31}C_{{\mathrm{OH}}^{-}}^{n32}$
4	${\mathrm{ZnOH}}^{+}+{\mathrm{OH}}^{-}\to \mathrm{Zn}{\left(\mathrm{OH}\right)}_{2}$	$k_{4}$	*1.9*	*0.4*	$k_{4}C_{{\mathrm{ZnOH}}^{+}}^{n41}C_{{\mathrm{OH}}^{-}}^{n42}$
5	$\mathrm{Zn}{\left(\mathrm{OH}\right)}_{2,\left(aq\right)}\to \mathrm{ZnO}+H_{2}O$	$k_{5}$	1.0	–	$k_{5}C_{\mathrm{Zn}{\left(\mathrm{OH}\right)}_{2\left(aq\right)}}^{n51}$

**Table 2:** Simulation model results for ZnO electrodeposition in pulsed −4 mA current at 60 °C, bubbling O_2_ and solution 0.1 M KCl, 5 × 10^−3^ M ZnCl_2_.

Time (s)	ZnOH^+^ (mN/L)	Zn(OH)_2_ (µm/cm^2^)	ZnO (µm/cm^2^)
0	5.83 × 10^−6^	0.000	0.000
20	8.46 × 10^−6^	0.182	0.000
40	4.60 × 10^−6^	0.381	0.000
60	8.28 × 10^−6^	0.598	0.000
80	1.24 × 10^−6^	0.702	0.014
100	1.64 × 10^−6^	0.702	0.015
120	2.31 × 10^−6^	0.702	0.032
140	2.61 × 10^−6^	0.702	0.054
–	–	–	–
680	5.83 × 10^−6^	0.702	7.091
700	5.83 × 10^−6^	0.702	7.114
720	5.83 × 10^−6^	0.702	7.133
740	5.83 × 10^−5^	0.702	7.156
760	5.83 × 10^−5^	0.702	7.176
780	5.83 × 10^−5^	0.702	7.195
800	5.83 × 10^−6^	0.702	7.210

**Table 3:** Crystallite sizes measured by the Scherrer equation and surface areas measured by the BET.

Property	ZnO
Crystallite size (nm)	10.2
Surface area (m^2^/g)	20.6

## Data Availability

Data presented in this article are available on request from the corresponding author.
